# Identification of possible hypoxia sensor for behavioral responses in a marine annelid, *Capitella teleta*

**DOI:** 10.1242/bio.037630

**Published:** 2019-02-11

**Authors:** Tetsuya Ogino, Haruhiko Toyohara

**Affiliations:** Division of Applied Biosciences, Graduate School of Agriculture, Kyoto University, Kitashirakawa-Oiwake-cho, Sakyo-ku, Kyoto, 606-8502, Japan

**Keywords:** Hypoxia, Marine annelid, TRP channel, Behavior

## Abstract

Hypoxia often occurs in summer and causes deleterious effects on marine benthic animals. A marine annelid, *Capitella teleta*, is tolerant to hypoxia, as shown by the fact that it inhabits organically polluted areas, where severe hypoxia is often observed. To understand how this species adapts to the environment, we focused on its hypoxia sensor, and we showed that TRPAbasal was a possible contributor to hypoxia detection in *C. teleta*. To examine the involvement of TRPA1 in the response of *C. teleta* to hypoxia, we exposed *C. teleta* to hypoxic water with or without a TRPA1-specific inhibitor, A-967079. Hypoxic stimulation induced escape behavior in *C. teleta* from the sediment, and this behavior was suppressed by the inhibitor. The cloned TRPA gene from *C. teleta* was phylogenetically categorized into *TRPAbasal*, and contains an oxygen-dependent degradation domain, which is important for the detection of hypoxia. Whole-mount *in situ* hybridization analysis showed that the gene was transcribed in the prostomium, where sensing functions are localized. These results suggested that the worm has a hypoxia-sensing system possibly utilizing CtTRPAbasal, and this system contributes to expanding the organism's niches in hypoxic environments by detecting whether hypoxia exceeds a level that would imperil its survival.

## Abstract (precis)

**Summary:** The hypoxia-avoidance behavior of a marine annelid was suppressed by a TRPA1-specific inhibitor. Our dataset suggests that the possible hypoxia sensor for hypoxia-inducing behaviors of this marine annelid is TRPAbasal.

## INTRODUCTION

Hypoxic zones are expanding in open oceans and coastal waters due to global warming and high nutrient inputs, and have influences on ecosystems and biodiversity (Breitburg et al., 2018; Diaz and Rosenberg, 2008; Keeling et al., 2010). This expansion is considered likely to continue, and to have unpredictable impacts on marine animals, including species commercially important for fishing. Hypoxia is conventionally defined as dissolved oxygen (DO) concentration <2 mg/l (Vaquer-Sunyer and Duarte, 2008). The effects of hypoxia vary depending on the species, although they are roughly similar within a particular taxonomic group, and reflect differences in animals' behavioral and physiological adaptability to hypoxia (Diaz and Rosenberg, 1995; Vaquer-Sunyer and Duarte, 2008). It is important to clearly understand the influence of hypoxia on marine benthic animals, because these animals have important roles in supporting marine ecology.

Marine annelids of the genus *Capitella* are small, thread-like worms with hypoxia tolerance (Rosenberg, 1972). They inhabit organically enriched sediment in coastal water, where severe hypoxia occurs in summer (Gray et al., 2002; Tsutsumi, 1987). Their population shows rapid growth prior to other species in autumn as DO rises (Méndez et al., 1997; Tsutsumi, 1987). Therefore, they are thought to respond sensitively to the fluctuation of DO and to return to the habitat rapidly as DO rises.

Responses of marine invertebrates to hypoxia can be simply categorized into physiological and behavioral responses (Breitburg et al., 2018; Wu, 2002). The physiological responses are promotion of hemoglobin synthesis (Gorr et al., 2004) and enhancement of the anaerobic metabolism (Albert and Ellington, 1985; Schöttler and Grieshaber, 1988; Stickle et al., 1989). Hypoxia inducible factors (HIFs) play regulatory functions in these responses, and their mechanism of regulation is well understood in terrestrial organisms (Gorr et al., 2006; Ke and Costa, 2006; Wang et al., 1995). The regulation of physiological responses by HIFs was also observed in marine animals such as bivalves, crustaceans and fish (Kotsyuba, 2017; Piontkivska et al., 2011; Soitamo et al., 2001; Li and Brouwer, 2007). On the other hand, the behavioral responses include avoidance of hypoxic water (Eby and Crowder, 2002; Wu et al., 2002) and suppression of heartbeat rates (Eby and Crowder, 2002; Marshall and McQuaid, 1993). However, there is little knowledge about the hypoxia sensing system that regulates behavioral responses in marine invertebrates.

Recently, in mammals, one member of the transient receptor potential (TRP) channel family, TRPA1, was reported to be involved in the hypoxia detection system and to regulate hypoxia-triggered behavioral responses (Pokorski et al., 2014; Takahashi et al., 2011). In this study, we showed that TRPA possibly contributes to the hypoxia-detection system of marine invertebrates, as indicated by our experiments on hypoxia-avoidance behavior of *C. teleta*.

## RESULTS

### Hypoxia avoidance assay

**Fig. 1.**
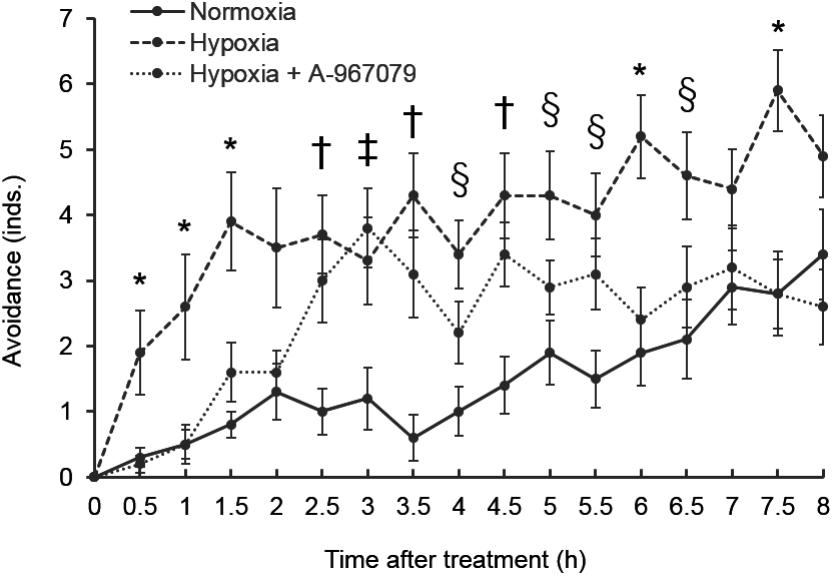
**Hypoxia avoidance assay.** Values are shown as mean±s.e.m. of the number of worms that protruded from the glass beads (*n*=10). Symbols within this figure indicate the statistically significant differences determined by ANOVA followed by Tukey's test (*P*<0.05) as follows: *, hypoxia versus normoxia or hypoxia+A-967079; †, normoxia versus hypoxia or hypoxia+A-967079; ‡, normoxia versus hypoxia+A-967079; §, hypoxia versus normoxia.

To examine the involvement of TRPA1 in hypoxia detection by *C. teleta*, the effects of a TRPA1-specific inhibitor, A-967079, on the hypoxia-induced response were examined (Fig. 1). The worms exposed to severe hypoxic conditions crawled out of the sediment, and the number of worms showing this behavior in severe hypoxia treatment was significantly larger than that in normoxia treatment at every time point examined except for 2, 3, 7 and 8 h. This behavior was suppressed by 10 µM A-967079 at 0.5, 1, 1.5, 6 and 7.5 h.

To determine the concentration of DO in the hypoxia avoidance assay, the same experiment as above without A-967079 treatment was conducted three times for measuring DO concentrations. The concentrations of DO in the normoxia treatment were 5.80, 5.91, 5.95 mg/l at 0 h, 2.95, 3.46, 3.60 mg/l at 4 h and 3.57, 3.68, 4.26 mg/l at 8 h. In the hypoxia treatment, the concentrations of DO were 0.39, 0.41, 0.46 mg/l at 0 h, 1.24, 1.66, 1.82 mg/l at 4 h and 1.71, 1.80, 1.87 mg/l at 8 h. Throughout this experiment, the temperature was in the range of 24–24.9°C.

### Effect of A-967079 on locomotor activity

To ensure that A-967079 had no side effect on the worms' locomotor activity, the migration distances (measured at the tip of their head) with or without A-967079 were evaluated (Fig. S1). The average migration distance (mm)±s.d. with A-967079 treatment was 103.6±48.7, while with the mock treatment it was 131.0±56.0. The *P*-value between the two treatments evaluated by Student's *t*-test was 0.22.

### Domain structure and phylogenic analysis of CtTRPAbasal

**Fig. 2.**
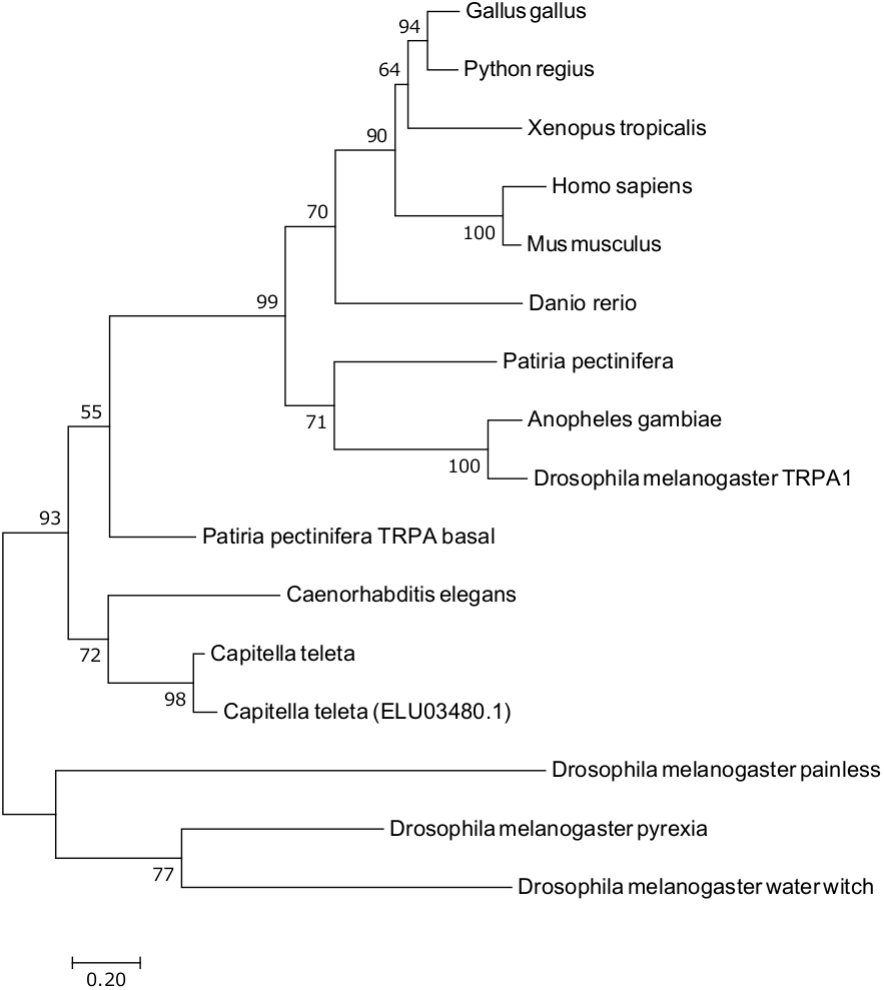
**Phylogenic position of CtTRPAbasal.** Phylogenic tree of known TRPA channels was constructed by the maximum likelihood method. Only transmembrane domains were used for constructing the tree. Bootstrap values calculated using 1000 replications are shown near the nodes. Gene accession numbers of the amino acid sequences used in this analysis are shown in Table S1.

**Fig. 3.**
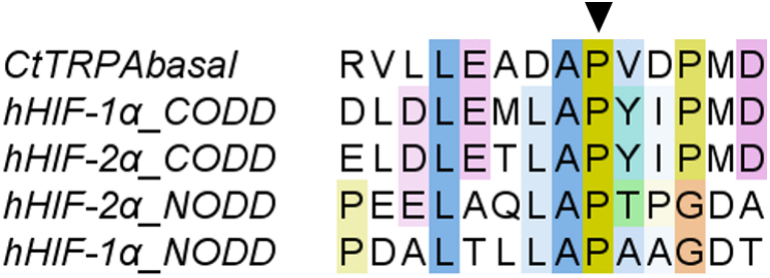
**Alignment of oxygen-dependent degradation domain of human HIFα with that of CtTRPAbasal.** The amino acid sequences of HIF-1a and 2a were acquired from NCBI, and the gene accession numbers were Q16665.1 and Q99814.3, respectively. Alignment was done using ClustalW services and the result was analyzed using Jalview. Coloring of amino acids was performed according to ClustalX. Arrowhead indicates the hydroxylation site.

To speculate about the function of CtTRPAbasal, its domain structure and phylogeny were examined. The sequence of CtTRPAbasal has been deposited in the DDBJ database under accession number LC462258. The phylogenic relationship of CtTRPAbasal with TRPA from various species from nematode to man was analyzed (Fig. 2). This analysis showed that CtTRPAbasal belongs to the same clade as nematode's TRPA and TRPAbasal found in the starfish *Patiria pectinifera*. Many ankyrin repeats in the N-terminal cytosolic region are a typical feature of TRPA, and CtTRPAbasal is speculated to have 16 ankyrin repeats there. The oxygen-dependent degradation domain (ODD) has an important role in the detection of hypoxia in mTRPA1 (Takahashi et al., 2011). To confirm the presence of ODD in CtTRPAbasal, amino acid sequences of ODDs in human HIF-1α and HIF-2α were aligned with CtTRPAbasal, and ODD was found in the N-terminal cytosolic region of CtTRPAbasal (Fig. 3).

### Whole-mount *in situ* hybridization

**Fig. 4.**
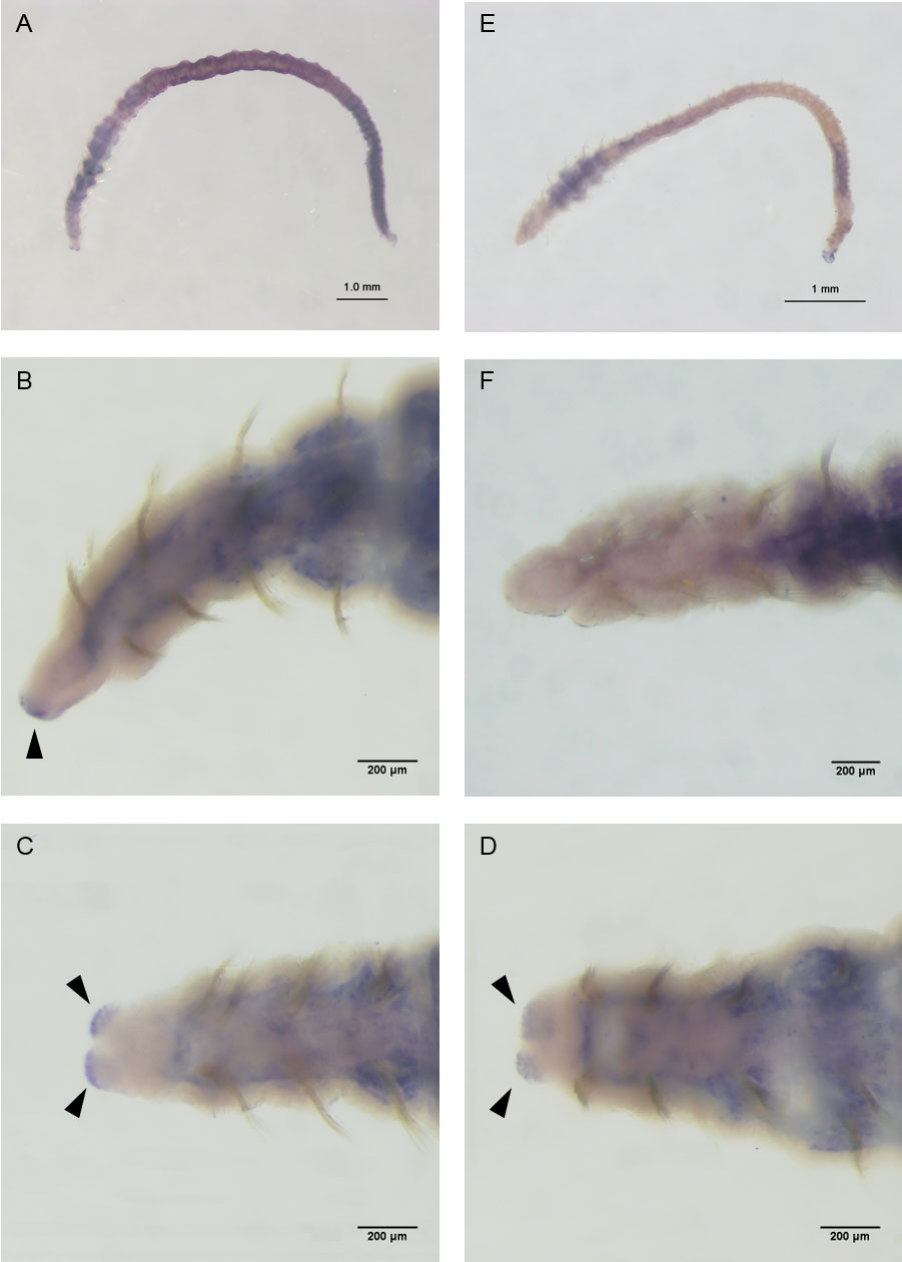
**Whole-mount *in situ* hybridization.** The probe consisted of 521 bp from bp 1945 to bp 2466 in the open reading frame of CtTRPA. Signals were developed using BM-Purple (blue). Arrowheads indicate the signals at the prostomium. (A) Whole body from lateral side with anti-sense probe. (B) Lateral side of the head with anti-sense probe. (C) Ventral side of the head with anti-sense probe. (D) Dorsal side of the head with anti-sense probe. (E) Whole body from lateral side with sense probe. (F) Lateral side of the head with sense probe.

To identify where in the worm CtTRPAbasal works, *in situ* hybridization analyses were performed. A strong, sharply defined signal was observed at the prostomium, and a vague signal was observed in the posterior region (Fig. 4). However, a sense probe used as a negative control also produced a signal in the posterior region. Therefore, the true signal was concluded to be at the prostomium. The signal in the prostomium was located on both sides as seen from the ventral view.

## DISCUSSION

Hypoxia treatment induced escape behavior of *C. teleta*, and this behavior was suppressed by the administration of A-967079 (Fig. 1), while the locomotor activity was not suppressed by A-967079 (Fig. S1). The suppression of escape behavior in *C. teleta* was prominent at the beginning of the experiment, which suggests that A-967079 influenced a rapid response to hypoxia. In mice, TRPA1 is the key regulator of the hypoxia ventilatory response, i.e. of the increase in ventilation during hypoxia (Pokorski et al., 2014). When mice breathe hypoxic air, their respiratory rate increases rapidly, but that of TRPA1-inhibited mice does not. This is in accord with our results, which implied that TRPA1 was involved in the rapid behavioral response to hypoxia. At later time points, A-967079 also suppressed the hypoxia-escaping behavior. Hatano et al., 2012 showed that human TRPA1 was upregulated within several hours by activated hypoxia inducible factor 1 α, a hypoxia-responsive transcription factor. This suggests that the input of sensing hypoxia via the TRPA1 homologue could gradually increase as the time of exposure to hypoxia increased. This might explain our observation that the suppression of the escape behavior re-emerged in the late part of this experiment. In the middle part of this experiment, this suppression was not observed. This might be because other hypoxia-sensing mechanisms, such as soluble guanylyl cyclase (sGC) or hypoxia inducible factor pathways, overwhelmed the A-967079-induced TRPA1 inhibition.

Similar hypoxia-induced evacuation from sediment is observed in infaunal benthic species. The burrowing marine annelids *Hediste diversicolor* and *Alitta virens* expose themselves on the sediment in response to hypoxia (Vismann, 1990). *Lomia medusa*, a tubicolous marine annelid, escapes from its tubes in hypoxic conditions (Llansó and Diaz, 1994). The brittle stars *Amphiura filiformis* and *A. chiajei*, the deposit-feeding bivalves *Abra alba* and *A. nitida*, and the suspension-feeding bivalve *Cerastoderma edule* come out from sediment in response to hypoxic stimuli (Rosenberg et al., 1991). In this study, *C. teleta* climbed on the sides of glass vials with their mucous in response to hypoxia. We infer that when they are confronted by hypoxia in their natural habitat, they would crawl out of their burrow and migrate on the surface of the sediment to find more suitable conditions. Terrestrial model organisms also show similar hypoxia-induced behaviors. The larvae of *Drosophila melanogaster* evacuate from their food, yeast paste, in response to hypoxia (Wingrove and O'Farrell, 1999). *Caenorhabditis elegans* migrates to an environment with a preferred oxygen availability to avoid hypoxia and hyperoxia (Gray et al., 2004; Chang and Bargmann, 2008). These behavioral responses are thought to be regulated by the nitric oxide (NO)/cyclic guanosine monophosphate (cGMP) signaling pathway. In this pathway, soluble guanylyl cyclases play a key role in sensing oxygen via NO production (Morton, 2004; Vermehren-Schmaedick et al., 2010). These cyclases produce cGMP in response to oxygen deprivation, and cGMP in turn activates cyclic nucleotide-gated ion channel (CNG). Activation of CNG allows calcium ions to pass through the plasma membrane, resulting in depolarization, which leads to the behavioral responses to hypoxia. Hypoxia-induced activation of TRPA1 homologue also increases calcium permeability. TRPA1 homologue as a hypoxia sensor may cooperate with sGC to promote escape from the sediment in hypoxic conditions.

The phylogenic analysis of the TRPA1 homologue gene suggested that the cloned TRPA gene from *C. teleta* was classified as a TRPAbasal gene, similar to nematode's TRPA-1 homologues (Fig. 2). The known agonists of nematode's TRPA-1 are cold (Xiao et al., 2013) and mechanical stimuli (Kindt et al., 2007). A starfish, *Patiria pectinifera*, also has two TRPAs, TRPA1 and TRPA basal (Saito et al., 2017). PpTRPA1 is thermosensitive and involved in thermotaxis, but PpTRPA basal is not activated by heat or several pungent chemicals. The ODD is important for activation of TRPA1 by hypoxia. In the normoxic condition, a proline residue in the ODD is hydroxylated by the PHD family, which are oxygen-dependent prolyl hydroxylases (Takahashi et al., 2011). Under hypoxic conditions, that proline is not hydroxylated, leading to activation of TRPA1. This oxygen-dependent hydroxylation was first found in the regulation of HIFα, which mediates the physiological response to hypoxia (Kaelin and Ratcliffe, 2008). Therefore, the ODD from HIF-1α was aligned with CtTRPAbasal to examine whether CtTRPAbasal possesses an ODD (Fig. 3). The results of alignment showed that CtTRPAbasal has an ODD in the N-terminal cytosolic region, like mTRPA1. This result suggested that CtTRPAbasal can be activated by hypoxia. On the other hand, online transcriptome data indicate the existence of a gene homologous to CtTRPAbasal, ELU03480.1, in *C. teleta*. This gene is also categorized into TRPAbasal (Fig. 2) but the proline residue in ODD is substituted by glutamine, which suggests that this gene is not involved in hypoxia detection (Fig. S2).

The whole-mount *in situ* hybridization analysis showed that *CtTRPAbasal* was transcribed precisely in the segment anterior to mouth called the prostomium (Fig. 4). In annelids, sensory cells for sensing environmental stimuli such as amino acids and pH are specifically localized in the prostomium (Lindsay et al., 2004; Laverack, 1960, 1961). Observation of the ventral view revealed that signals were observed in both sides of the prostomium. In the tip of the prostomium, nerve fibers are concentrated on both lateral sides (Meyer et al., 2015). Since the arrangement of nerve fibers is similar to the location of the *CtTRPAbasal* transcript signal there, CtTRPAbasal may function as a hypoxia sensor at the sensory cells in the prostomium. Therefore, *C. teleta* might sense DO in its direction of movement and effectively avoid hypoxic zones. To specify more precisely the localization of CtTRPAbasal protein, immunohistochemical analyses will be needed.

A-967079 antagonizes mammalian TRPA1 with high specificity. Nakatsuka et al. (2013) and Banzawa et al. (2014) showed that A-967079 does not antagonize, but instead activates, chicken and frog TRPA1. This difference of antagonistic or agonistic effects of A-967079 depends on several amino acids present in the 5th transmembrane region. The region containing these amino acids in CtTRPAbasal does not have high similarity with that in mammalian TRPA1. Therefore, there is a possibility that *C. teleta* has some other TRPA1 homologue with higher homology to mammalian TRPA1, and A-967079 has no antagonistic effect on CtTRPAbasal. On the other hand, CtTRPA1 contains an ODD, a key domain for hypoxia-activation, suggesting that CtTRPAbasal would regulate the hypoxia-induced response of *C. teleta*. The suppression of hypoxia-avoidance behavior of *C. teleta* by A-967079 supports the notion that A-967079 antagonizes CtTRPAbasal activation by hypoxia. Functional analyses of CtTRPAbasal itself will be needed to verify the hypoxia-sensing ability of CtTRPAbasal and the antagonistic effect of A-967079 on it.

To expand their habitats, it is important for organisms to sense the limit of their tolerance to physicochemical conditions for their survival. Organisms belonging to the *C. capitata* complex, including several capitellids of which *C. teleta* is one, can endure several days or more in severe hypoxic conditions (Warren, 1977). *Capitella teleta* is thought to decrease its aerobic metabolism to below approximately 1.5 mg/l to endure hypoxic conditions, based on the results of its oxygen uptake rate (Chareonpanich et al., 1994). These reports showed the high tolerance of *C. teleta* to hypoxia. In this study, *C. teleta* exhibited avoidance from severe hypoxia, possibly mediated by CtTRPAbasal, within 1 h, and hence they could migrate to an area with higher DO before the time limit for their survival in severely hypoxic conditions. Their behavioral response and tolerance to hypoxia led them to survive in niches in organically enriched sediments where hypoxia often occurs. However, whether CtTRPAbasal itself is activated by hypoxia needs to be clarified by loss-of-function methods or analysis of the ion channel function.

## MATERIALS AND METHODS

### Rearing of *C. teleta*

Stock cultures of *C. teleta* were kindly provided by Dr H. Tsutsumi of Prefectural University of Kumamoto and Dr N. Ueda of the University of Kitakyushu, and reared in mud with artificial sea water (ASW) (Rei-sea marine II, Iwaki, Tokyo, Japan) of 33 psu salinity at 18°C. The worms were originally collected from the sediment below a fish farm (32°23′52 N, 130°13′40 E) or Dokai Bay (33°52′34 N, 130°45′3 E). Worms were fed commercial fish food.

### Hypoxia avoidance assay

10 ml of glass beads (BZ-01, AS ONE Corporation, Osaka, Japan) and 50 ml of ASW were poured into glass vials (No.7L, AS ONE Corporation). Ten bare worms were transferred into the vials and kept for more than 3 h at room temperature. Stock solutions of 100 mM A-967079 (Wako Pure Chemical Industries, Ltd., Osaka, Japan) were made in DMSO. 5 μL of A-967079 stock solution was added to the ASW in each glass vial containing worms, making a final concentration of 10 μM, while in control treatments, DMSO alone was added. To ensure the inhibitory effect of A-967079, these vials were kept for 10 min. Hypoxia was achieved by blowing nitrogen gas into the solution for 10 min. Then the cap was closed and vials were allowed to stand for 8 h, during which photographs were taken every 30 min (Movie 1). Hypoxia-avoidance behavior was evaluated by the number of worms of which partly protruded from the glass beads. This experiment was repeated ten times. Real-time monitoring of DO could not be done to avoid disturbing the environmental condition in the vial. Therefore, the same experiments as above without A-967079 treatment were conducted only to measure DO. Dissolved oxygen concentrations at 0 h, 4 h and 8 h were measured using a Seven2Go DO meter (Mettler Toledo, Columbus, USA). The measurements were repeated three times.

### Effect of A-967079 on the locomotor activity

The bare worms were rinsed with ASW and transferred to a dish with a diameter of 8.75 cm containing 25 ml ASW supplemented with 0.01% 100 mM A-967079 dissolved in DMSO (final 10 µM A-967069) or 0.01% DMSO as mock control. Photographs were taken with a WG-5 GPS camera (Ricoh, Tokyo, Japan) at the rate of one photograph/min for 42 min immediately after transfer. The coordinates of the head of worms were estimated using ImageJ (Schneider et al., 2012). Their migration distance during 30 min was calculated to determine the effect of A-967079 on their movement activity. This analysis was done using the 11th to 41st photograph because the first 10 min was taken as the time to ensure the inhibitory effect of A-9670679. The number of replications of A-967079 treatment was 12, while that of the mock control was 13.

### Cloning, domain structure and phylogenetic analysis of CtTRPAbasal

A part of the TRPA gene sequence from *C*. *teleta* was acquired by BLAST search using the human TRPA1 protein sequence (NP_0015628.2) as query. The Sequence ID of the most similar protein sequence is ELT91340.1. The sequence of full-length CtTRPAbasal was determined by the rapid amplification of cDNA ends method, and the open reading frame with flanking regions was cloned with two primers containing restriction enzyme sites at the 5′ends; Fw: ACTAAAGCTTTAACGCTGCATCAGTGCGCTCG, Rv; ACTAGGATCCTAACCAGACAACGGCTTGAAAC. This was amplified using KOD Plus Neo (TOYOBO, Osaka, Japan) with the following program; initial incubation at 94°C for 2 min; 35 cycles of denaturation at 98°C for 10 s, annealing at 60°C for 30 s and amplification at 68°C for 3.5 min, and final incubation at 68°C for 5 min. Amplified products were cut with *Bam*HI and *Hind*III and inserted into pBluescript KS(-).

The domain structure of CtTRPAbasal was analyzed using InterProScan (Jones et al., 2014). Phylogenic analysis was performed using MEGA7 (Kumar et al., 2016). Gene IDs used for phylogenic analysis of CtTRPAbasal are listed in Table S1. Transmembrane regions in the deduced amino acid sequence of these genes were determined using Pfam search (Finn et al., 2016) and aligned by using MUSCLE (Edgar, 2004). A phylogenic tree was constructed by the maximum likelihood method using the LG model. A discrete gamma distribution was used to model evolutionary rate differences among sites [five categories (+*G*, parameter=1.3532)]. The gaps were completely deleted. The ODD domain of CtTRPAbasal was deduced by alignment with the ODD domain from human HIF-1α and HIF-2α (Masson et al., 2001) using ClustalW (Larkin et al., 2007). Analysis of the alignment was performed using Jalview (Waterhouse et al., 2009).

### Whole-mount *in situ* hybridization

Worms were kept in a 0.5% agar plate buffered with ASW for 2 days to remove the mud inside the gut. Then, the worms were relaxed in 1:1 0.37 M MgCl_2_:ASW for more than 10 min. Relaxed worms were fixed with 4% paraformaldehyde (PFA)/PBS overnight at 4°C. Dehydration was achieved as follows: incubation in 25% methanol/75% PBS for 5 min, 50% methanol/50% PBS for 5 min, 75% methanol/25% PBS for 5 min, 100% methanol twice for 10 min each, and the dehydrated samples were then stored at −20°C until experiments.

Samples were rehydrated with 75% methanol/25% PBST (PBS, 0.1% Tween 20) for 5 min, 50% methanol/50% PBST for 5 min, 25% methanol/75% PBST for 5 min and three times with PBST for 5 min each, and then digested with protease K (10 μg/ml) in PBST for 30 min at room temperature. After washing with PBST, samples were soaked in 0.1 M triethanolamine (pH 7–8) for 5 min to inactivate endogenous alkaline phosphatase. Then the samples were transferred to fresh 0.1 M triethanolamine and 1/400 volume of acetic anhydride was added to block the nonspecific binding of probes. 5 min later, another 1/400 volume of acetic anhydride was added and the samples were incubated for 5 min. Then, samples were washed two times with PBST for 5 min and fixed with 4% PFA for 1 h followed by three washes with PBST for 5 min. Samples were washed with hybridization buffer (Hyb; 50% formamide, 5×SSC, 50 μg/ml heparin, 0.1% Tween 20, 1% SDS, 100 μg/ml ribonucleic acid from torula yeast), and then prehybridized with fresh hybridization buffer for 2 h at 65°C. Digoxigenin-labeled RNA probes were synthesized using a DIG RNA Labeling Kit (Roche, Basel, Switzerland) (with T3 for antisense probe and T7 for sense probe). The template was 521 bp from 1945 to 2466 bp in the open reading frame of CtTRPAbasal cloned into pBluescript KS (−). Hybridization was carried out using probes at 0.1 ng/μl overnight at 65°C. After hybridization, worms were washed with Hyb for 5 min and then for 20 min, two times with 75% Hyb/25% PBST for 20 min, two times with 50% Hyb/50% PBST for 20 min and two times with 25% Hyb/75% PBST for 20 min at 65°C, and then three times with PBST for 5 min and five times with PBT (1×PBS, 0.2% TritonX-100, 0.1% BSA). Riboprobes were visualized as follows: samples were incubated for 1 h with blocking buffer [1×blocking buffer (Roche), PBS (−)], then overnight with anti-Digoxygenin Fab Fragment (Roche) diluted 1:5000 in blocking buffer at 4°C, and then washed three times with PBT for 5 min. Color development was carried out using BM-Purple (Roche). Colored samples were then equilibrated in 80% glycerol/1×PBS and images were captured with VB-7000 (Keyence, Osaka, Japan) mounted on MZFLIII (Leica Microsystems, Wetzlar, Germany). Image stacking of multiple focal planes was performed with ImageJ.

## Supplementary Material

Supplementary information
