# Supplementary material for: Identification of possible hypoxia sensor for behavioral responses in a marine annelid, Capitella teleta
Source: Biol Open. 2019 Feb 11;8(3):bio037630. doi: 10.1242/bio.037630 (PMC6451338; doi:10.1242/bio.037630)
Supplement: Supplementary information [file biolopen-8-037630-s1.pdf]

Supporting information

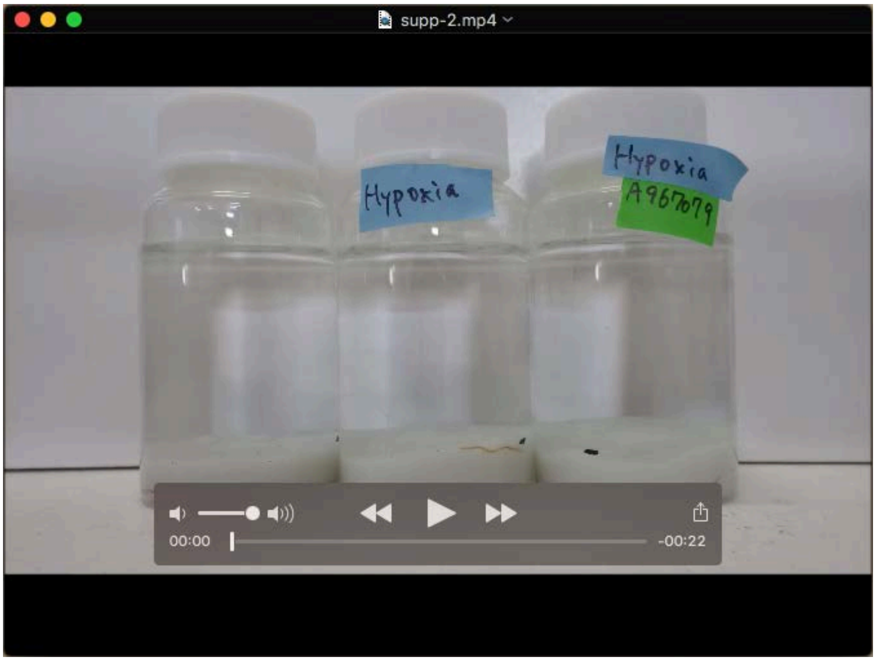

Movie 1. Hypoxia avoidance assay. Left: normoxia, middle: hypoxia, right: hypoxia + A-967079.

Biology Open • Supplementary information

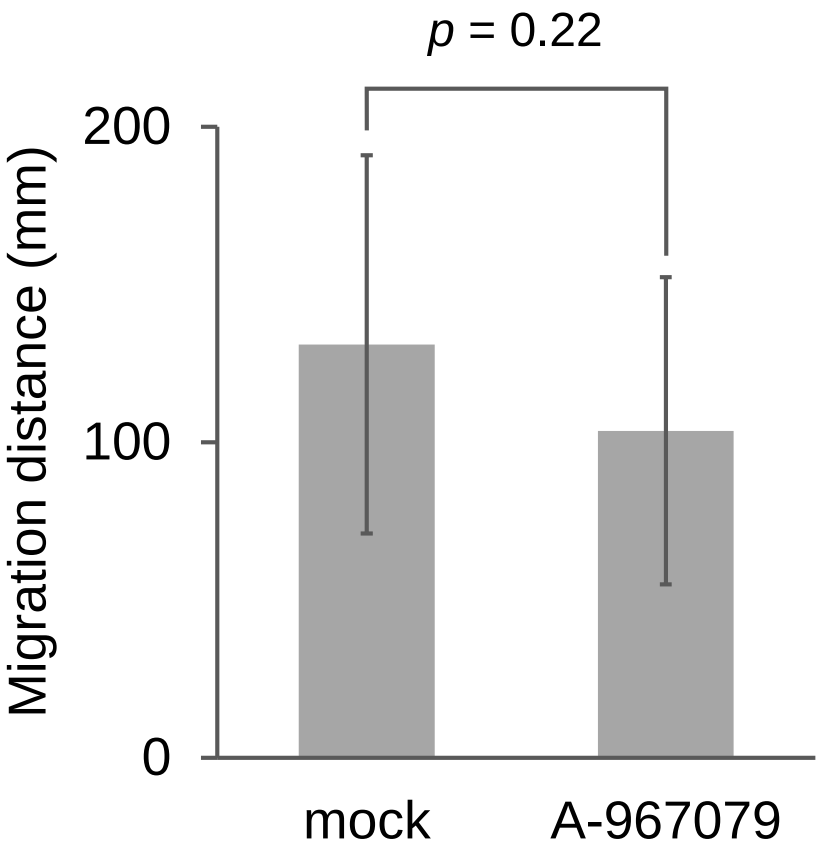

Fig. S1. Effect of A-967079 on the locomotor activity. Migration distance of the head of *Capitella teleta* for 30 min is shown when they were immersed in artificial seawater with (n = 12) or without 10 μM A-967079 (n =13). Data are shown as mean ± s.d. The data of the two groups were statistically analyzed by Student's *t*-test.

Biology Open • Supplementary information

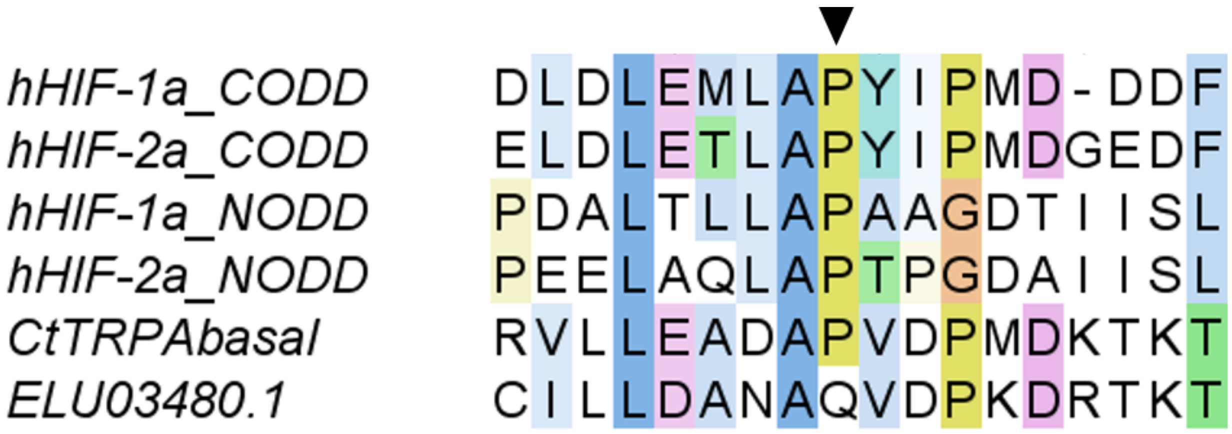

Fig. S2. Alignment of two CtTRPAbasal genes with ODDs in human HIFα. Alignment was performed using ClustalW. Jalview was used for analysis of this alignment. Arrowhead indicates hydroxylation site.

Biology Open • Supplementary information

Table S1. Gene accession number used for phylogenic analysis.

Sequence name in the tree	Accession number
Caenorhabditis elegans	NP_502249.3
Drosophila melanogaster painless	NP_611979
Drosophila melanogaster pyrexia	NP_612015
Drosophila melanogaster water witch	NP_731193
Drosophila melanogaster TRPA1	NP_648263
Danio rerio	XP_009296845
Gallus gallus	BAO51998
Homo sapiens	NP_015628
Mus musculus	NP_808449
Python regius	ADD82928
Xenopus tropicalis	NP_001121434
Anopheles gambiae	ACC86138
Patiria pectinifera TRPA basal	BAX76613.1
Patiria pectinifera TRPA1	BAX76612.1
